# Complete Genome Characterization of Chinese Porcine Deltacoronavirus Strain CHN/Tianjin/2016

**DOI:** 10.1128/genomeA.00237-17

**Published:** 2017-04-20

**Authors:** Feng Zhao, Ying Sun, Bingxu Qian, Xiaorong Zhang, Yantao Wu

**Affiliations:** a College of Veterinary Medicine, Yangzhou University, Yangzhou, Jiangsu, China; b Jiangsu Co-Innovation Center for Prevention and Control of Important Animal Infectious Diseases and Zoonoses, Yangzhou, Jiangsu, China

## Abstract

As we all know, porcine deltacoronavirus was first detected in Hong Kong, China. Here, we report the complete genome sequence of the Chinese porcine deltacoronavirus strain CHN/Tianjin/2016, which was collected and amplified from clinical fecal samples in March of 2016.

## GENOME ANNOUNCEMENT

Porcine deltacoronavirus (PDCoV) belongs to the new group of coronaviruses called deltacoronaviruses (1). PDCoV, with a genome length of approximately 25 kb, is an enveloped, single-stranded, and positive-sense RNA virus. PDCoV was first detected from fecal samples in 2009 in Hong Kong during a molecular surveillance study in 2012 (2) and reported in the United States during an outbreak in swine populations in February 2014. The virus has spread to 17 U.S. states since then (3). Although PDCoV was first identified in Hong Kong, there is no evidence that the existence of PDCoV in mainland China has a genetic relationship with U.S. PDCoV (4).

In March 2016, our laboratory received several fecal samples from pigs that had clinical signs of diarrhea. Molecular biology detection indicated that the samples did not contain porcine epidemic diarrhea virus, transmissible gastroenteritis virus, or porcine rotavirus. All of the samples were positive for PDCoV by real-time reverse transcription-PCR (RT-PCR), with cycle threshold (*C*_*T*_) values around 25. To better understand the genetic characteristics of PDCoV from mainland China, the complete genome sequence of the PDCoV CHN/Tianjin/2016 strain was amplified using a previously described method (3).

The genomic sequence of the PDCoV CHN/Tianjin/2016 strain is 2,5413 nucleotides (nt) in length, excluding the 3′ poly(A) tail. The genomic structure of the PDCoV CHN/Tianjin/2016 strain contains a 5′ untranslated region (UTR), a 3′ UTR, and eight open reading frames (ORFs). Among the eight ORFs, four coding structural proteins, namely, spike (S), envelope (E), nucleocapsid (N), and membrane (M) proteins, were found; the other four coding nonstructural proteins, namely, ORF 1a/1b, nonstructural protein 6 (NS6), and nonstructural protein 7 (NS7), were also found.

The full-length genomic sequence analysis of the PDCoV CHN/Tianjin/2016 strain showed that it had the highest nucleotide identity (99%) with Chinese epidemic strains (GenBank accession numbers KP757892, KP757891, KR131621, KT266822, and JQ065043, etc.) and some U.S. PDCoV strains which were mostly detected in 2014 (GenBank accession numbers KJ769231, KJ601780, KJ567050, and KR265850, etc.).

The PDCoV CHN/Tianjin/2016 strain’s sequence data will facilitate future research on the epidemiology and evolutionary biology of PDCoV in China. Also, the sequence supplied by our laboratory can promote understanding of the genetic relationship with other PDCoV strains reported in the United States, South Korea, and Hong Kong.

### Accession number(s).

The complete genome sequence of PDCoV strain CHN/Tianjin/2016 has been deposited in GenBank under the accession number KY065120.

## References

[1] De Groot RJ, Baker SC, Baric R, Enjuanes L, Gorbalenya AE, Holmes KV, Perlman S, Poon L, Rottier PJM, Talbot PJ, Woo PCY, Ziebuhr J 2011 Coronaviridae, p 806–828. *In* King AMQ, Adams MJ, Carstens EB, Lefkowitz EJ (ed), Virus taxonomy: ninth report of the International Committee on Taxonomy of Viruses. Elsevier Academic, London, United Kingdom.

[2] Woo PC, Lau SK, Lam CS, Lau CC, Tsang AK, Lau JH, Bai R, Teng JL, Tsang CC, Wang M, Zheng BJ, Chan KH, Yuen KY 2012 Discovery of seven novel mammalian and avian coronaviruses in the genus *Deltacoronavirus* supports bat coronaviruses as the gene source of alphacoronavirus and betacoronavirus and avian coronaviruses as the gene source of gammacoronavirus and deltacoronavirus. J Virol 86:3995–4008. doi:10.1128/JVI.06540-11. 22278237 PMC3302495

[3] Wang L, Byrum B, Zhang Y 2014 Detection and genetic characterization of deltacoronavirus in pigs, Ohio, USA, 2014. Emerg Infect Dis 20:1227–1230. doi:10.3201/eid2007.140296. 24964136 PMC4073853

[4] Li G, Chen Q, Harmon KM, Yoon KJ, Schwartz KJ, Hoogland MJ, Gauger PC, Main RG, Zhang J 2014 Full-length genome sequence of porcine deltacoronavirus strain USA/IA/2014/8734. Genome Announc 2(2):e00278-14. doi:10.1128/genomeA.00278-14. 24723718 PMC3983307

